# A Linear, Millimetre Displacement-to-Frequency Transducer

**DOI:** 10.3390/s120810820

**Published:** 2012-08-06

**Authors:** John T. Agee, Four K. Petto

**Affiliations:** 1 Department of Electrical Engineering, Tshwane University of Technology, Private Bag X680, Pretoria 0001, South Africa; 2 P.O. Box 401783, Broadhurst, Gaborone, Botswana; E-Mail: pettok@bpc.bw

**Keywords:** millimetre displacement, displacement-to-frequency, high-fidelity

## Abstract

The paper presents a novel linear, high-fidelity millimetre displacement-to-frequency transducer, based on the resistive conversion of displacement into a proportional voltage, and then frequency. The derivation of the nonlinearity, fidelity and sensitivity of the transducer is presented. Experimental results confirm that a displacement of 0–100 mm is converted into a frequency range of 0–100 kHz, with a normalised fidelity factor of 99.91%, and a worst-case nonlinearity of less than 0.08%. Tests using laboratory standards show that a displacement of 10 mm is transduced with an accuracy of ±0.6%, and a standard deviation of 530 Hz. Estimates included in the paper show that the transducer could cost less than 1% of existing systems for millimeter displacement measurement.

## 1. Introduction

Several ultrasound, optical or laser-based devices exist for the measurement of displacements larger than one metre [1–4]. The cost of the modifications required for the use of these systems for measuring displacements in the range of a few micrometres to millimetres (submetre) is only justifiable in a few circumstances. For affordable submetre displacement measurements, capacitive and inductive position sensors are often used. However, the frequency dependence of capacitive and inductive sensors limits their domains of application [5–7]. In fact, a comparative discourse relating the range of displacement measurable *versus* the sensor recommended, could be found in [8].

In process and industrial instrumentation systems, several variables are detected using elastic sensors as primary sensing elements. Elastic sensors often generate displacements in the range of several micrometres to millimeters, which have to be conditioned further. Table 1, derived from information available in Chapter 8 of [9], shows example applications of elastic sensors resulting in an intermediate displacement variable. Moreover, physiological changes in biological tissues resulting from dehydration, accumulation of fluid due to disease, *etc.*, can be studied using submetre displacement measurements [10]. Millimeter displacement is also encountered in the analysis of the integrity of civil structures [11,12], where such measurement systems as the GPS-RTS are currently used. A key challenge in the current systems for millimeter-displacement measurement is the high cost of acquisition of such measurement systems. Hence, there is significant motivation for the exploration of cheaper systems for use in small displacement measurement. Moreover, the transducers most suitable for the conditioning of such small displacement signals must have high sensitivity, high fidelity and minimum nonlinearity for acceptable accuracy of transduction. This paper presents the design, analysis and experimental validation of a submetre displacement-to-frequency transducer. The system is based on the sensitivity of some resistive elements to displacement. Resistive sensors are relatively cheap; and their zero-order dynamics make them suitable for both static and dynamic measurements. Unlike time-of-flight devices or phase-based measurement systems, resistive millimeter displacement transducers need be coupled physically to the displacement being measured.

In the rest of the paper, the circuit design, analysis of the basic displacement-to-voltage converter, and the implementation of the primary conditioning amplifier circuit are presented in Section 2. The voltage-to-frequency conversion design is presented in Section 3 of the paper. Circuit realisation, experimental results, and discussions of these results form Section 4 of the paper. Section 5 presents conclusions and the limitation of the transducer circuit. A list of references concludes the paper.

## 2. The Basic Displacement Sensor, and the Design and Analysis of the Primary Conditioning Circuit

**Figure 1.**
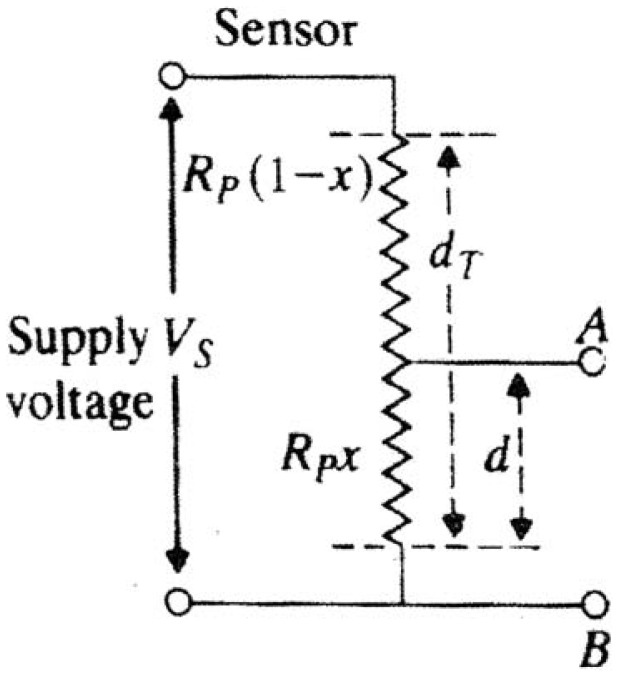
Arrangement of potentiometer-type displacement sensor.

The basic displacement-to-voltage sensor is shown in Figure 1. The sensor consists of a three-terminal potentiometer of total resistance R_P_, supplied by a DC voltage V_s_. The resistance between terminals A and B of the potentiometer is directly related to the displacement *d(t)* (alternatively, the normalised displacement *x*), where $0\leq x=\frac{d}{d_{T}}\leq 1;$ and *d(t)* = *xd*_T_. Then:

(1) $$\begin{array}{cc} \frac{E_{TH}}{V_{s}}=\frac{R_{p}x}{R_{p}} & or \end{array}E_{TH}=V_{s}x$$

Note that, the maximum value of E_TH_ is V_s_, when x = 1. The Thevenin's resistance of the equivalent sensor circuit, *R_TH_*, is evaluated to yield [9]:

(2) $$R_{TH}=\frac{R_{p}(1-x)R_{p}x}{R_{p}(1-x)+R_{p}x}=R_{P}x(1-x)$$

The sensitivity of **E_TH_** to the normalised displacement **x** is given by:

(3) $$S_{E_{TH}}^{x}=\frac{\partial E_{TH}}{\partial x}=V_{s}$$

To avoid excessive power dissipation in the resistance of the potentiometer, V_s_ is usually kept small.

Consequently, the sensitivity $S_{E_{TH}}^{x}$ of this basic sensor is small. Additional conditioning is required to improve the sensitivity of the sensor. Now, the normalised sensitivity is given by:

(4) $$S_{\mu E_{TH}}^{x}=\frac{1}{V_{s}}\frac{\partial E_{TH}}{\partial_{x}}=1$$

### 2.1. Primary Conditioning of Sensor Output

**Figure 2.**
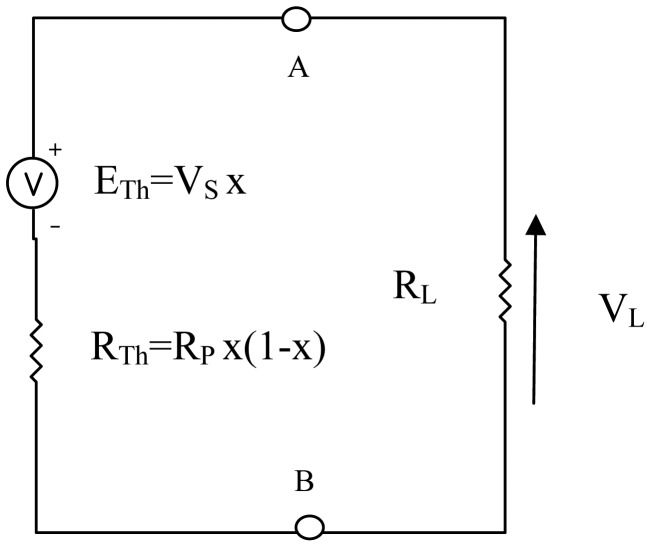
Equivalent circuit of sensor with primary conditioning amplifier.

The equivalent circuit resulting from the connection of a primary amplifier of input resistance R_L_, across terminals AB of the sensor circuit is shown in Figure 2. Loading effects tend to degrade the performance of amplifiers. The loading effect of the conditioning circuit modifies the Thevenin's voltage to:

(5) $$V_{L}=\frac{R_{L}}{R_{L}+R_{TH}}\cdot V_{s}x$$

The normalised value of this voltage is also derived to be:

(6) $$V_{L\mu }=\frac{V_{L}}{V_{s}}=\frac{R_{L}x}{R_{L}+R_{P}x(1-x)}$$

In the next sub-section of the paper, Equations (5) and (6) are used to analyse the quality of the displacement-to-voltage conversion amplifier, and to show any additional condition(s) that could be imposed on the conditioning circuit to further improve the performance of the transducer.

### 2.2. Quality Analysis of the Primary Conditioning Circuit

In this subsection, the analysis of the quality of the primary signal amplification, based on Equation (5), is presented.

#### 2.2.1. Fidelity of Primary Amplifier

Fidelity is a measure of how faithfully a circuit has processed a given signal to minimize distortions. The concept of fidelity is usually used in the analysis of high frequency amplifiers. In the current paper, the concept of fidelity is used to quantify the loading effect of the primary conditioning amplifier on the signal produced by the sensor. Now, the voltage drop due to the loading effect of R_L_ is obtained to be:

(7) $$\Delta V=E_{TH}-V_{L}=\frac{R_{P}(x^{2}-x^{3})}{R_{L}+R_{P}(x-x^{2})}V_{s}$$

or, in normalised form:

(8) $$\Delta V_{\mu }=\frac{\Delta V}{V_{s}}=\frac{R_{P}(x^{2}-x^{3})}{R_{L}+R_{P}(x-x^{2})}$$

The normalised fidelity factor is then given as:

(9) $$F_{\mu }=1-\Delta V_{\mu }=\frac{1+Kx{\left(1-x\right)}^{2}}{1+Kx(1-x)}$$

For perfect fidelity, *K* = 0. Practically, this requires that:

(10) $$K=\frac{R_{P}}{R_{L}}\to 0;R_{L}\to \infty$$

#### 2.2.2. Sensitivity Analysis

The sensitivity of the conditioned output is derived to yield:

(11) $$S_{V_{L}}^{x}=\frac{\partial V_{L}}{\partial x}=\frac{R_{L}^{2}+R_{L}R_{P}x^{2}}{{\left[R_{L}+R_{P}x(x-x^{2})\right]}^{2}}V_{s}$$

The normalised sensitivity is found to be:

(12) $$S_{V_{L\mu }}^{x}=\frac{1+Kx^{2}}{{\left[1+K(x-x^{2})\right]}^{2}}$$

It is required to select the value of K in such a manner, as to minimize variations $S_{V_{L\mu }}^{x}$ within the range of measurements.

#### 2.2.3. Nonlinearity Effects

From Equation (5)*V_L_* is nonlinear in ***x***. The nonlinearity N(x) can be quantified by using:

(13) $$V_{L}=m^{*}x+c^{*}+N(x)$$

where the linear part of *V_L_* is defined by the following parameters:

(14) $$\begin{array}{l} m^{*}=\frac{V_{L}(x_{\max})-V_{L}(x_{\min})}{x_{\max}-x_{\min}}V_{S}\\ c^{*}=V_{L}(x_{\min})-m^{*}x_{\min};x_{\max}=1,x_{\min}=0,c^{*}=0 \end{array}$$

and the nonlinearity *N(x)* is given as:

(15) $$\begin{array}{l} ∴N(x)=V_{s}x-m^{*}x-c^{*}\\ N(x)=V_{s}\left\{\frac{Kx^{2}(1-x)}{1+Kx(1-x)}\right\}\\ N_{\mu }(x)=\frac{Kx^{2}(1-x)}{1+Kx(1-x)} \end{array}$$

Nonlinearity is not desirable, and is eliminated as in Equation (10). In fact, it is evident from Equations (9), (10) and (15) that *K* → 0 improves linearity and fidelity. This contradicts the requirement for enhanced sensitivity as in Equation (12), for which *K* → ∞.The approach in this paper is to select *K* → 0 for fidelity and linearity enhancement; and to effect sensitivity improvement using voltage to frequency conversion.

#### 2.2.4. Practical Realisation of the Signal Conditioning Amplifier

**Figure 3.**
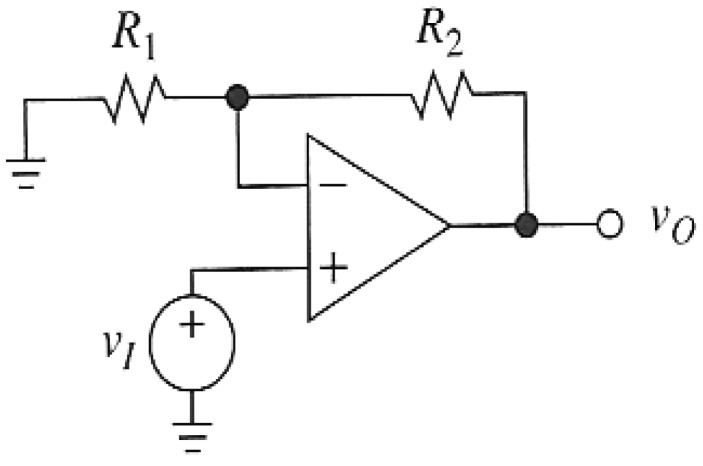
Summing amplifier used as the primary conditioning circuit.

The practical implementation of the primary conditioning amplifier uses the summing amplifier shown in Figure 3 [13,14], with the amplified voltage given by:

(16) $$\begin{array}{c} v_{0}=(1+R_{2}/R_{1})V_{L}\\ =(1+R_{2}/R_{1})\frac{V_{s}}{1+Kx(1-x)}x\\ =(1+R_{2}/R_{1})\frac{V_{s}}{1+Kx(1-x)}\frac{d(t)}{d_{T}} \end{array}$$

An amplifier gain of 10 was used for the current work. This yields the normalised sensitivity parameter given in Equation (17).

(17) $$S_{v_{0}}^{x}=\frac{10(1+Kx^{2})}{{\left[1+K(x-x^{2})\right]}^{2}}$$

Table 2 summarises the parameters of the sensor and the amplifying circuit.

Combining the parameter values in Table 2 with Equations (5) and (10) yields:

(18) $$0.998x\leq V_{L}=\frac{V_{S}}{1+0.0056x(1-x)}x\leq 1.006x$$

Therefore, within an accuracy of ±0.6%:

(19) $$V_{L}=x[V]$$

Similarly:

(20) $$\begin{array}{cc} v_{0}=10V_{L}=10x=10\frac{d(t)}{d_{T}}[V];d(t),d_{T} & \left[mm\right] \end{array}$$

and the sensitivity of the amplified voltage is given by:

(21) $$S_{v_{0}}^{d}=0.1[V/mm];d_{T}=100mm$$

In Section 3, we present a technique to further improve the sensitivity of the transducer, using voltage-to-frequency conversion.

## 3. Sensitivity Enhancement

As observed above, a small value of K (0.0056) was required to both minimize nonlinearity effects, and to enhance fidelity of the primary conditioning circuit. This value of K however, lowers the sensitivity of the transduction process. Since submetre displacements can be very small, a very high sensitivity transducer is required (as shown in Table 2, ideal sensitivity required is ∞). In the sequel, we present a voltage-to-frequency converter circuit that is used to further enhance the sensitivity of the developed transducer.

### 3.1. Voltage-to-Frequency (VFC) Conversion

Apart from sensitivity enhancement, the conversion of *v*_0_ into a frequency signal has several other advantages, including: high noise immunity, high output power, wide dynamic range, and ease of interfacing with digital data acquisition systems. Table 3 shows key values of *v*_0_ and their corresponding frequency representations.

The linear relationship between v_0_ and frequency in Table 3 is expressed mathematically as:

(22) $$f_{0}=10000v_{0}[Hz]$$

Applying Equation (20) in Equation (22) we obtain:

(23) $$\begin{array}{ccc} f_{0}=1000d(t) & \left[Hz];d(t\right) & \left[mm\right] \end{array}$$

It is evident from Equation (23) that:

(24) $$\begin{array}{ccc} d(t)=\frac{f_{0}}{1000} & [mm];f_{0} & \left[Hz\right] \end{array}$$

The AD 650 voltage-to-frequency converter (VFC) was used for the implementation of the displacement-to-frequency conversion circuit satisfying Equation (23). The pin layout of the AD 650 VFC is obtained from the manufacturer's manual for the device [15]. The selection of components for the VFC circuit is presented in the sequel.

### 3.2. Component's Selection for the AD 650 VFC

For the AD 650, only four component values must be selected by the user [15]. Using the manufacturer's notation, these are the input resistance R_IN_, the timing capacitor C_OS_, the logic resistor R_2_ and the integration capacitor C_INT_. The first two are determined by the input voltage range and full-scale frequency. Additional relationship between R_IN_ and C_OS_ is provided through graphs obtainable in [15]. Sample design for a maximum frequency of 100 KHZ in the data sheet of the AD 650 VFC used R_IN_ = 40 kΩ and this has been adopted for the realization in this study. Table 4 summarizes the components used for the design of the VFC circuit, with C_INT_ calculated using the equation:

(25) $$C_{\text{INT}}=\frac{10^{-4}F/\sec}{f_{\text{MAX}}}\geq 1000pF$$

The frequency conversion improves the sensitivity of the transducer from the value given by Equation (17) to:

(26) $$S_{v_{0}}^{Hz}=\frac{100000-0}{\left(100-0\right)}=10^{3}\text{bits}/mm$$

and has also improved the resolution to = 10^−3^ mm/bit.

## 4. Simulations, Experimental Validation, Results Presentation and Discussions

The indices assessing the quality of the transducer were evaluated by simulation. The rest of the results were obtained through experimental measurements.

### 4.1. Simulations

**Figure 4.**
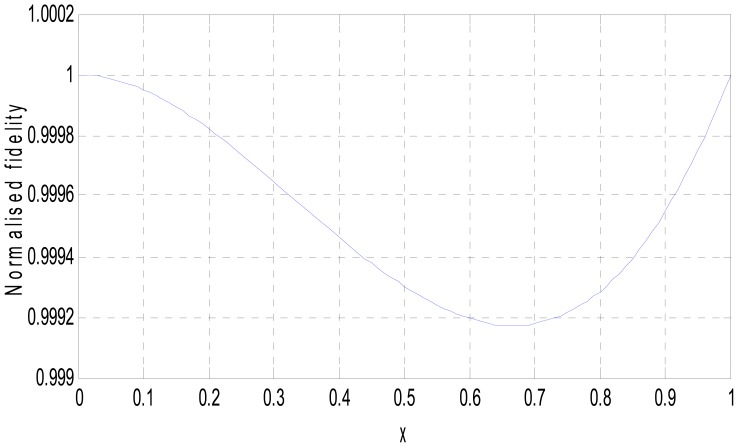
Fidelity of primary voltage conditioning amplifier.

**Figure 5.**
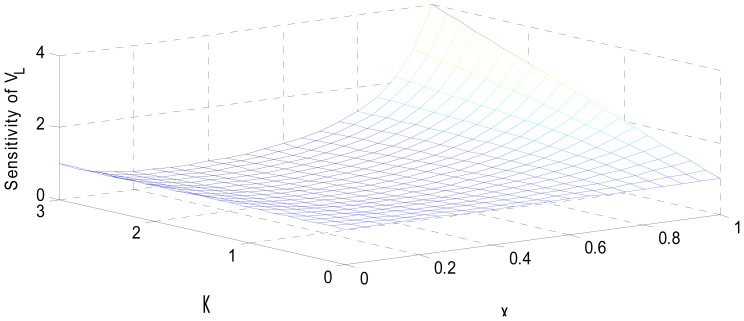
Sensitivity analysis in displacement-to-voltage conversion.

**Figure 6.**
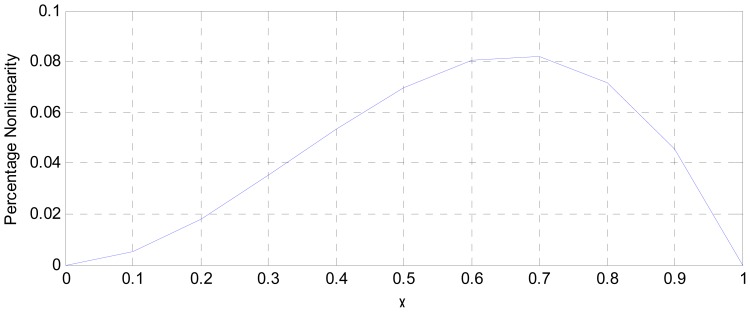
Analysis of nonlinearity in displacement to voltage conversion.

MATLAB simulation of the transducer fidelity, sensitivity and nonlinearity, based on Equations (9), (12) and (15) is presented in Figures 4–6.

### 4.2. Experimental Validation

**Figure 7.**
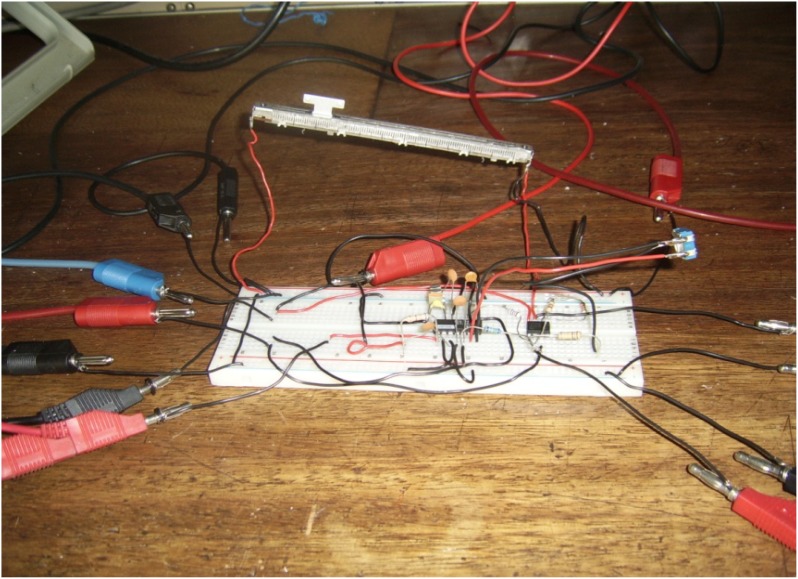
Experimental setup of transducer.

**Figure 8.**
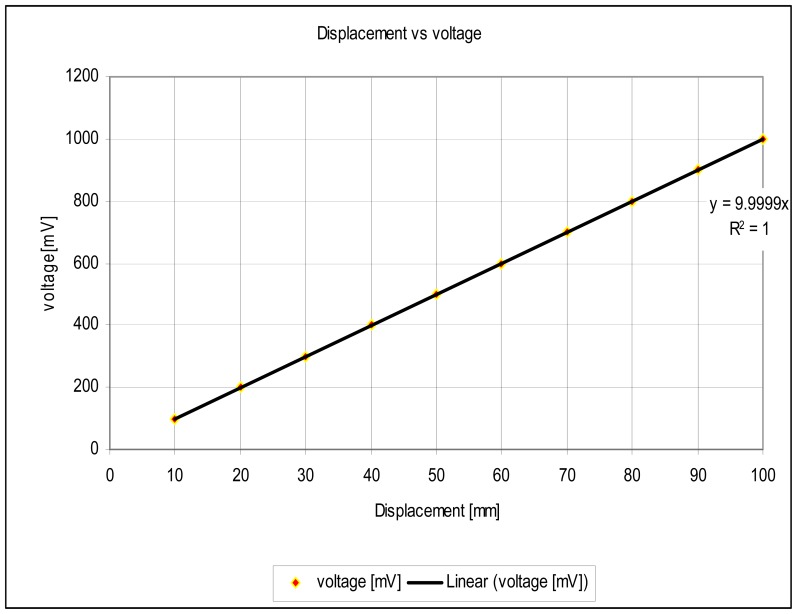
Basic sub-meter-to-voltage conversion.

**Figure 9.**
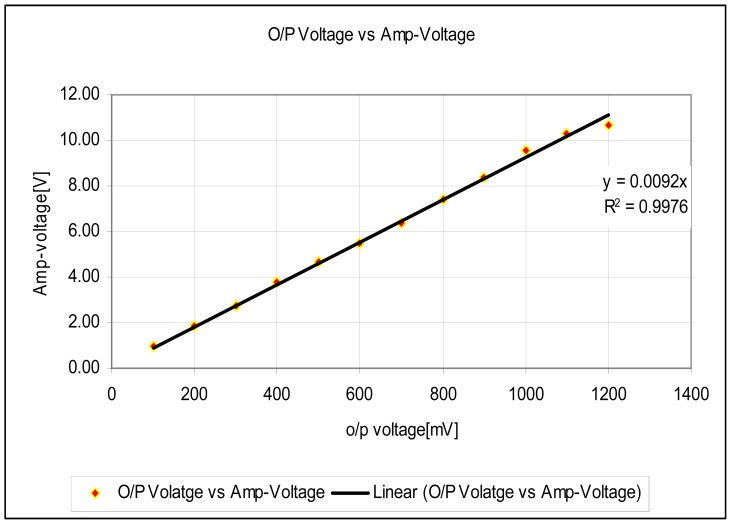
Performance of voltage amplifier.

**Figure 10.**
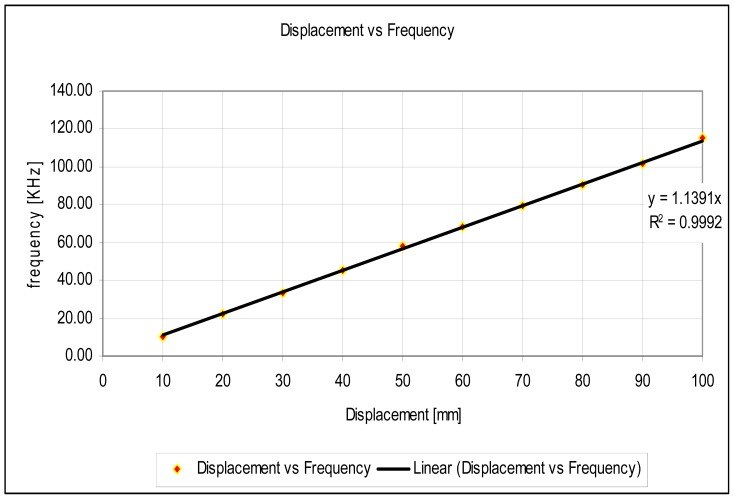
Displacement to frequency transduction.

**Figure 11.**
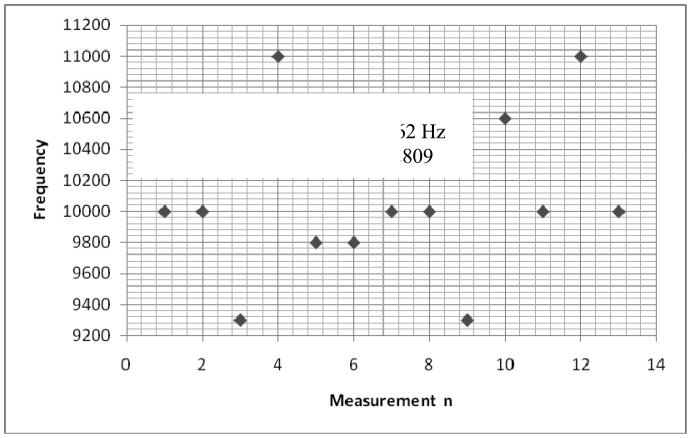
Sample measurements from transducer for precision analysis.

**Figure 12.**
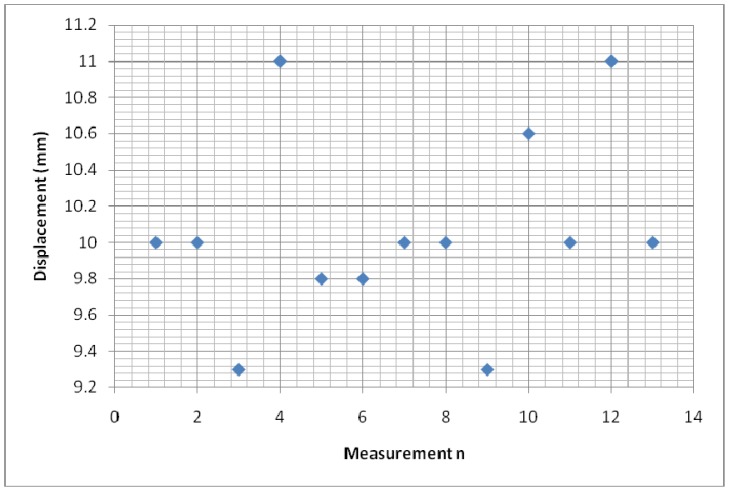
Equivalent millimeter output from frequency measurements.

The experimental setup is shown in Figure 7. For the experiments, a slide wire potentiometer was used as the submeter displacement sensor. It had a maximum displacement d_T_ = 100 mm = 10^−1^ m, and a total resistance of 11.2 kΩ (instead of the design maximum resistance of 10 kΩ). The potentiometer was supplied by a 1volt DC supply. The Thevenin voltage of the sensor, as a function of displacement, is shown in Figure 8. A plot of the amplified sensor voltage as function of detected displacement is shown in Figure 9. The overall displacement-to-frequency transduction is shown in Figure 10. For the analysis of the accuracy and precision of transducing displacement inputs into frequency, repeated measurements of 10 mm displacement were undertaken. The results are shown in Figures 11 and 12.

### 4.3. Result Discussions

The simulated fidelity results presented in Figure 4 shows that, the conditioning of the sensor signal by the primary amplifier was undertaken with at least 99.91% fidelity. Figure 5 shows that the introduction of the conditioning circuit slightly reduced the normalized input sensitivity $\left(S_{\mu E_{th}}^{x}\right)$ to a value less than unity (0.988). As shown in Figure 6, the choice of K = 0.0056 has reduced the worst case nonlinearity to 0.18% < 4%, as typically allowed in instrumentation [9]. The experimental measurements, confirmed the linearity between the displacement and the displacement-dependent voltage E_TH_ as in Figure 8. Figure 9 shows that, the connection of the voltage amplifier has introduced nearly 0.22% nonlinearity (obtained from the linear correlation coefficient); also slightly reducing the normalized sensitivity from a value of unity to 0.9978. Experimental results of the overall displacement-to-frequency conversion process are shown in Figure 10. It is evident there-from, that the millimetre-to-frequency converter has an overall linearity to 99.92%, or a nonlinearity of 0.08%. Results from the precision analysis of the displacement-to-frequency transducer are shown in Figures 11 and 12. It is evident from these, that for a 10 mm displacement, a mean measurement of 10,062 Hz (for 100,000 Hz) was obtained, giving a transducer accuracy of ±0.62%; the standard deviation of the measurements was 530 Hz.

### 4.4. Cost-Analysis

The reported millimeter-to-frequency transducer consists of one resistive sensor, five resistors, two capacitors, two operational amplifiers and one AD 650 voltage-to-frequency converter. Table 5 shows the cost estimation for the new transducer based on average component cost, and 30% device production fee. Calculations are shown for two sensor types: potentiometers and strain gauges.

To put the above costs in perspective, Table 6 compares the cost of the reported transducer with those of existing displacement sensors.

It is evident from Table 6, that the reported transducer has a very significant financial advantage over several existing systems for displacement measurements.

## 5. Conclusions

It is concluded that a cheap, linear, millimetre displacement-to-frequency transducer with both high sensitivity and high fidelity has been successfully realised.

### Limitations

The design sensor resistance of 10 kΩ was not available. A sensor of total resistance of 11.2 kΩ was used instead. Whereas this larger resistance value did not directly affect the accurate performance of the transducer, it was observed that, the maximum output frequency was 120 kHz (instead of the design maximum frequency of 100 kHz). Temperature variations constitute a significant random impact on sensor performance. Temperature effects have not yet being characterized. The effect of supply voltage variation is also still under investigation. Test measurements were undertaken using laboratory standards. Traceability of accuracy shall be undertaken in subsequent development, using facilities at a national metrology centre.

## Figures and Tables

**Table 1.** Example of elastic sensors producing submtre displacement as output [9].

**Elastic Sensor**	**Primary Variable**

Strain gauge	Force
Parallel plate capacitor	Pressure
Diaphragms	Pressure
Bellows	Pressure
Bourdon tubes	Pressure
Load cells	Pressure
Cantilever sensors	Force
Cylindrical shafts	Torque
Proving ring	Force

**Table 2.** System parameters.

**Parameter**	**Design Value**	**Ideal/Used Value**

d_T_	100 mm	
R_P_	10 kΩ	11.2 kΩ
R_L_	2 MΩ	∞
R_1_	20 kΩ	
R_2_	180 kΩ	
V_s_	1.0 V	
K = R_P_/R_L_	0.0056	0
N_μ_(x)	0≤ N_μ_(x) ≤ 0.00083	0
*F_μ_*	0.9992 ≤ *F_μ_* ≤ 1.000	1
$S_{V_{L\mu }}^{x}$	$0.9981\leq S_{V_{L\mu }}^{x}\leq 1.0056$	∞

**Table 3.** Voltage-to-frequency table.

**d (mm)**	**x**	***v*_0_ (V)**	**Frequency (kHz)**

0	0	0	0
100	1	10	100

**Table 4.** Parameters of the voltage-to-frequency converter.

**Parameter**	**Value**

f_max_	100 kHz
R_in_	40 kΩ
C_INT_	1,000 pF
C_OS_	330 pF
R_2_	1.75 kΩ

**Table 5.** Cost analysis per unit of the millimeter-to-frequency transducer.

Potentiometer sensor: $11.0/unit						$48.70
Components	5× Resistors: $7.50	2× Capacitors: $6.0	2× Op Amp: $2.0	1× AD 650: $11.00	Labour costs at 30%	Total transducer cost
Strain gauge: $3.50/unit						$39.00

**Table 6.** Cost comparison with some existing position sensors

**Sensor**	**Cost**	**Source of Information**

Potentiometer millimeter-to-frequency converter	$39,00	Table 5 in the paper
Strain gauge type Millimeter-to-frequency transducer	$48.7.00	Table 5 in the paper
SwissRanger 4000 (SR4000)	$9,000	http://www.hizook.com/blog/2010/03/28/low-cost-depth-cameras-aka-ranging-cameras-or-rgb-d-cameras-emerge-2010
PMD Technologies CamCube 2.0	$12,000	http://www.hizook.com/blog/2010/03/28/low-cost-depth-cameras-aka-ranging-cameras-or-rgb-d-cameras-emerge-2010
Hewlett Packard model 5525A Laser system	$11,500	http://www.n4mw.com/hp5526/hple.htm
